# mHealth Applications in Saudi Arabia: Current Features and Future Opportunities

**DOI:** 10.3390/healthcare13121392

**Published:** 2025-06-11

**Authors:** Sultan A. Alharthi

**Affiliations:** Department of Software Engineering, College of Computer Science and Engineering, University of Jeddah, Jeddah 21959, Saudi Arabia; saalharthi8@uj.edu.sa

**Keywords:** mHealth, healthcare, telemedicine, accessibility, user-centered design

## Abstract

**Introduction:** The rapid growth of mobile health (mHealth) applications has revolutionized healthcare delivery worldwide. These digital tools encompass a broad array of functionalities, including telemedicine, appointment scheduling, medication management, and health data tracking, all of which contribute to enhanced healthcare accessibility, increased patient engagement, and improved operational efficiency. However, despite their increasing prominence, the design, deployment, and use of mHealth applications continue to face several challenges, such as usability issues and overall sustained adoption. **Objectives:** This study aims to evaluate mHealth applications in Saudi Arabia, focusing on their design characteristics, usability features, and current feature gaps. **Method:** A total of 21 mHealth applications were selected and analyzed using a thematic analysis approach. The apps were selected based on usage popularity in the Saudi market and relevance to national digital health strategies. Data were drawn from publicly available app store information, official app documentation, and expert evaluations. **Results:** The findings reveal that while mHealth applications excel in areas such as telemedicine, appointment booking, and health education, there are notable gaps in features such as behavior modification, patient monitoring, and health management. **Conclusions:** This study contributes to the growing body of research on mHealth by offering grounded insights into the functional landscape of digital health tools in Saudi Arabia. It also outlines practical recommendations to enhance usability, feature diversity, and alignment with evolving healthcare needs in Saudi Arabia and beyond.

## 1. Introduction

Mobile health (mHealth) applications have emerged as integral components of contemporary healthcare systems, offering innovative mechanisms to support patient care, health monitoring, and the promotion of overall wellness [1,2]. These digital tools encompass a broad array of functionalities, including telemedicine, appointment scheduling, medication management, and health data tracking, all of which contribute to enhanced healthcare accessibility, increased patient engagement, and improved operational efficiency [3]. The adoption of mHealth applications experienced significant acceleration during the COVID-19 pandemic, serving as essential instruments for maintaining the continuity of care amid widespread restrictions on in-person healthcare services [4,5]. Nevertheless, despite their increasing prominence, the design, deployment, and sustained use of mHealth applications continue to face several challenges [6,7]. Key issues such as ensuring usability, refining feature sets, and addressing user-centered requirements persist as substantial obstacles to their overall effectiveness [3,8].

Although mHealth applications have advanced considerably in improving healthcare accessibility, substantial disparities in healthcare outcomes continue to exist. Al-Hanawi [9] examines these disparities in Saudi Arabia, revealing pronounced inequities in health status. The study reports a higher incidence of poor health among individuals aged 70 and above, particularly those suffering from chronic illnesses and those with lower educational background or limited income. Moreover, the research highlights the influential roles of marital status, health insurance coverage, and broader socio-economic conditions in shaping these persistent healthcare inequalities.

Despite growing global interest in mHealth technologies and their demonstrated utility during health crises such as the COVID-19 pandemic, there remains a limited understanding of how these applications have evolved over time in Saudi Arabia. While numerous studies have assessed user perceptions, adoption drivers, and usability challenges with mHealth applications in Saudi Arabia (e.g., [7,10,11,12,13,14,15]), few have conducted a comprehensive evaluation of mHealth applications. There is a notable gap in the literature regarding the longitudinal progression of application features, the alignment of these features with emerging healthcare needs, and the extent to which lessons learned during the pandemic have informed subsequent design and functionality. Furthermore, existing studies tend to focus on user attitudes or general adoption metrics, with insufficient attention paid to the systematic assessment of application content and feature completeness. This lack of granular analysis limits the ability of developers to make informed decisions about how to improve mHealth systems for broader accessibility, equity, and long-term sustainability.

The current study further investigates the evolution of mHealth applications in Saudi Arabia before, during, and after the COVID-19 pandemic, exploring how this critical period influenced their adoption and feature development. To achieve these objectives, this research is guided by three research questions:

RQ1: What are the main features of mHealth applications available in Saudi Arabia?
RQ2: What are the current feature gaps and future opportunities in these applications?
RQ3: What are the design and practical implications that can be derived?

To address our research questions, we conducted a review of 21 widely used mHealth applications in Saudi Arabia (Table 1). This review focused on identifying strengths, which can be defined as the presence of features that offer significant user value in terms of utility, accessibility, or engagement, alongside recurring design patterns and notable limitations in implementation and functionality. The collected data were systematically analyzed using a thematic analysis approach, which allowed us to uncover key themes related to the usability, gaps, and opportunities within the current mHealth landscape. This method provided a structured framework for interpreting the findings and linking them to practical recommendations for improving mHealth solutions. The findings reveal that while mHealth applications excel in areas such as telemedicine, appointment booking, and health education, there are notable gaps in features such as behavior modification, patient monitoring, and health management. These insights provide a foundation for guiding designers, developers, and healthcare providers toward designing more comprehensive and user-centric mHealth solutions.

## 2. Background

mHealth applications have demonstrated a pivotal role during the COVID-19 pandemic by delivering essential healthcare services, including contact tracing, symptom monitoring, and remote consultations. These technologies facilitated real-time surveillance, thereby supporting timely public health interventions and effective resource distribution [4,5]. Applications such as *Tawakkalna* [13], *Health Code* [16], and *NHS COVID-19* [17] emerged on a global scale, employing geolocation technologies to detect transmission chains and track the spread of the virus. In the context of Saudi Arabia, several studies have investigated the adoption and effectiveness of these applications. Alghareeb et al. [8] highlighted the critical roles of usability and privacy in enhancing user satisfaction and the acceptance of mHealth solutions. Similarly, Almufarij and Alharbi [6] identified user trust in application security and awareness of functional features as key factors influencing adoption. Furthermore, Alanzi et al. [18] emphasized the effectiveness of the *Mawid* application in mitigating the burden on physical healthcare services during the pandemic.

Conversely, Alajwari et al. [19] identified demographic variables, such as age, educational attainment, and digital literacy as significant factors shaping public perceptions of telemedicine and mHealth applications. These findings highlight the importance of implementing targeted awareness campaigns and educational programs to address disparities in access and engagement. Furthermore, Hidayat-ur-Rehman et al. [13] confirmed the effectiveness of mHealth technologies, while emphasizing the need for broader public awareness to enhance adoption rates. Similarly, Alsahli and Hor [12] discussed the essential role of mHealth applications in improving healthcare delivery during the COVID-19 pandemic in Saudi Arabia, while also identifying key challenges such as limited usability and accessibility. Although these studies contribute valuable insights into the adoption of mHealth, they primarily concentrate on general user perceptions, offering limited analysis of specific application features or the current state of technological development.

Furthermore, research within the Human–Computer Interaction (HCI) and Computer-Supported Cooperative Work (CSCW) domains has examined the social and privacy-related implications of mHealth applications deployed during the COVID-19 pandemic, stressing the critical need to balance public health surveillance with the preservation of individual privacy rights (e.g., [20,21,22,23,24,25]). Hofmann et al. [25] illustrate how grassroots initiatives showcased the capacity of collective community action to alleviate resource scarcities during the pandemic. In a related vein, Alaqra and Kitkowska [22] investigate how internal drivers, such as feelings of social isolation and fear induced by the pandemic profoundly shaped individuals’ willingness to engage with mHealth technologies. Simultaneously, Seberger and Patil [20] examine the inherent tensions between safeguarding privacy and advancing the public good in the context of mHealth application adoption.

Additionally, Alanzi [3] conducted an evaluative study on user satisfaction with mHealth applications in Saudi Arabia. The findings indicated that while usability aspects, such as ease of learning, information retrieval, and interface design were positively perceived, notable issues remained concerning navigation consistency and overall functionality. The study also observed that the accelerated uptake of mHealth solutions was primarily driven by necessity amid the pandemic, rather than by intrinsic user engagement, thereby raising concerns about continued use in the post-pandemic context where traditional healthcare services are once again accessible. In a complementary perspective, Khamaj and Ali [26] contend that the effectiveness of mHealth applications hinges on inclusive design practices that address accessibility limitations, particularly among older populations. They advocate for enhanced usability to ensure equitable access and to mitigate disparities stemming from digital literacy gaps. Concurrently, Hadwan et al. [2] emphasize the indispensable role of user feedback in refining mHealth platforms in Saudi Arabia. Furthermore, Alzghaibi [27] explored the barriers to the adoption of the Sehaty application in Saudi Arabia. The study revealed that technical challenges, usability issues, and privacy concerns significantly affect user satisfaction and engagement. These studies together identify considerable usability and accessibility challenges, underlining the necessity of designing mHealth applications that effectively accommodate a diverse range of user requirements.

While existing research has examined the perceived usability and user experience of telehealth systems in Saudi Arabia, particularly in the post-COVID-19 context, important analytical gaps remain concerning the evolution of mHealth applications over time. For instance, Sayed et al. [28] conducted a cross-sectional assessment of telehealth usability, demonstrating widespread adoption and satisfaction among adult users. Their findings revealed high usability scores for key features such as ease of use, interface quality, and future use intent. However, significant challenges persist, especially in terms of reliability, and disparities were observed across demographic groups including educational level and geographic location. Despite providing critical insight into usability metrics, the study focused primarily on users’ current perceptions and did not trace the development of specific application features over the course of the pandemic.

The relationship between technology and health literacy has been examined in prior research [29,30,31]. These studies collectively demonstrate that limited technological literacy constitutes a significant barrier to the adoption of mHealth solutions. Smith and Magnani [30] investigated the convergence of mHealth and digital health literacy, highlighting the potential of mHealth platforms to facilitate improved self-management among individuals with chronic conditions. While the promise of mHealth technologies, such as wearable devices and mobile health applications in improving health outcomes is evident, the study also draws attention to enduring challenges, particularly inequities in digital health literacy. Populations deemed vulnerable, including older adults and individuals with limited technological proficiency, continue to encounter substantial obstacles in accessing and effectively utilizing mHealth services. Similarly, Triana et al. [31] examined the accelerated integration of telehealth during the COVID-19 pandemic and identified technological literacy as a key impediment to its effective implementation. The study found that many patients, especially older individuals, experienced difficulties operating digital devices, installing necessary software, and engaging in virtual consultations, factors that frequently led to interruptions in the continuity of care. These prior studies demonstrate that the use of mHealth applications is deeply interwoven with social, cultural, political, and psychological dimensions, all of which significantly influence both individual and collective attitudes toward their adoption. Collectively, these insights provide a foundational rationale for the present study and inform the development of more effective mHealth applications that align with diverse user needs.

The World Health Organization (WHO) has developed a standardized classification framework for digital health interventions to support the systematic analysis and integration of digital tools into health systems [32]. This taxonomy organizes digital functionalities into four key categories: interventions for clients, healthcare providers, health system managers, and data services. By applying this framework, researchers and policymakers can assess the roles, maturity, and alignment of digital solutions within a broader health infrastructure [33,34]. In this study, the WHO classification provides a structured lens through which to evaluate the functionalities of mHealth applications commonly used in Saudi Arabia, enabling a clearer understanding of these apps.

To systematically explore these multidimensional features, this study adopts a thematic analysis approach as its primary method [35]. Thematic analysis is a widely used method for identifying, analyzing, and reporting patterns or themes within qualitative data. It allows researchers to move beyond surface-level observations and uncover underlying meanings in the data. This method has been widely adopted in prior studies examining digital tools, games, and user experiences [36]. By following this method, this study ensures a structured, transparent approach to qualitative analysis, producing insights that are both rigorous and grounded in the empirical data.

## 3. Methodology

This study conducted a review of mHealth applications in Saudi Arabia, focusing on their features, accessibility, and evolution before, during, and after the COVID-19 pandemic (Table 1). This review aimed to evaluate key aspects such as usability, interface design, accessibility options, and the alignment of app functionalities with the diverse needs of healthcare users. To identify mHealth applications for review, we accessed a publicly available list of government-owned mHealth applications used in Saudi Arabia.Additional searches were conducted on the iOS App Store and Google Play Store using keywords such as *“health”*, *“hospital”*, *“clinic”*, and *“healthcare”*. These keywords ensured the inclusion of widely used mHealth applications catering to both public and private healthcare sectors. The combination of these approaches allowed for the identification of a dataset of mHealth applications that reflected the breadth of services available in Saudi Arabia.

### 3.1. Application Selection Criteria

After compiling an initial pool of 29 applications, inclusion and exclusion criteria were applied to refine the dataset. The inclusion criteria required applications to be explicitly designed for Saudi Arabia and for healthcare purposes, such as telemedicine, appointment booking, medication management, or health education. Additionally, the applications needed to be operational, available for download during the study period, and relevant to healthcare delivery in Saudi Arabia. Conversely, the exclusion criteria eliminated applications that were no longer operational, had been removed from app stores, or had been merged with other platforms to form super-apps, leading to redundancy (e.g., mHealth applications *Mawid*, *Seha*, and *Tetamman* were all combined to create a super-app named *Sehhaty*). Applications lacking sufficient healthcare functionalities or relevance to the study objectives were also excluded. This process resulted in the elimination of 8 applications, leaving a final dataset of 21 operational mHealth applications for detailed analysis (Table 1).

### 3.2. Data Analysis Procedures

Data from the app reviews were analyzed to identify the primary features of mHealth applications in Saudi Arabia and to examine their functional evolution. The evaluations were conducted by bilingual researchers fluent in both Arabic and English, with professional backgrounds in mHealth and UX design. This ensured accurate interpretation of app content, interface design, and functionality across both languages. Following the review process, all data were imported into ATLAS.ti (https://atlasti.com (accessed on 10 April 2025)), a qualitative data analysis software, to facilitate systematic coding and thematic development. A thematic analysis approach was employed to identify recurring patterns and categories related to design features and functional gaps [35]. The analysis followed a structured process:

- **First**, we familiarized ourselves with the data and generated initial codes by categorizing meaningful excerpts that reflected recurring features, designs, or concerns;
- **Second**, we organized these codes into preliminary themes, grouping them based on conceptual similarity and practical relevance to digital health delivery;
- **Third**, we reviewed and refined these themes by resolving overlaps and ensuring each theme was conceptually distinct and accurately represented the coded data;
- **Finally**, we analyzed the refined themes and synthesized them into structured feature categories that informed our subsequent evaluation of each application.

Our thematic analysis yielded an initial inductive set of 24 core features that emerged from repeated patterns observed across the 21 apps. These features were grounded in actual implementations and reflected common design and functionality trends within the Saudi mHealth ecosystem.

### 3.3. Feature Refinement and WHO Classification Mapping

**Figure 1:**
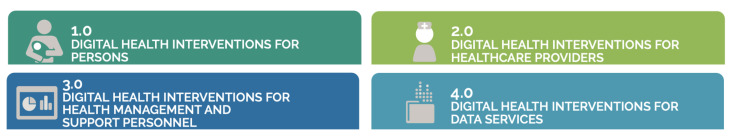
Overview of the different dimensions outlined in the World Health Organization (WHO) Classification of Digital Interventions, Services, and Applications in Health, which provides a standardized framework for categorizing digital health functionalities across client services, healthcare providers, health system management, and data services to support global health objectives [32].

To ensure completeness and alignment with international digital health standards, the initial feature set was then refined through a deductive process using the World Health Organization’s (WHO) Classification of Digital Interventions, Services, and Applications in Health [32]. This framework is widely used in prior studies [33,34,37], and allowed us to validate and expand our list by identifying essential and critical features that provide comprehensive mHealth functionality (Figure 1). By leveraging this classification, we were able to validate the inductively derived features and identify additional essential elements that are critical for delivering comprehensive, user-centered mHealth functionality.

**Figure 2:**
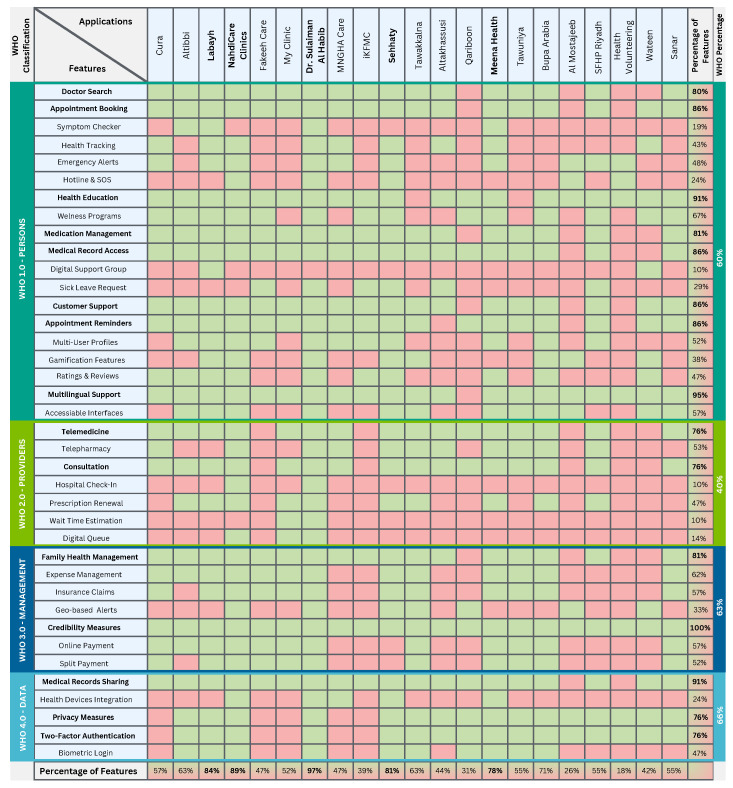
Feature coverage matrix of mHealth applications in Saudi Arabia. This matrix visualizes the presence or absence of 38 core features, mapped to the WHO Classification of Digital Interventions across 21 mHealth applications. Green cells indicate features that are implemented in each application; red cells highlight features that are absent; and coverage percentages are displayed along the margins. Applications and features displayed in bold represent those with 75% or higher coverage.

The integration of inductive and deductive approaches resulted in a final set of 38 unique features, combining empirical insight with theoretical rigor. Each of the 21 applications was then re-evaluated against this expanded list to assess the presence or absence of each feature. The outcome was a feature coverage matrix (Figure 2), which captures the breadth and distribution of functionalities across the sampled mHealth applications. This hybrid approach ensures that our findings are both grounded in real-world application practices and aligned with global digital health priorities.

## 4. Findings and Discussion

Before the COVID-19 pandemic, mHealth applications in Saudi Arabia focused primarily on core healthcare services (Table 1). Applications like *Cura*, *Altibbi*, and *Labayh* catered to niche services like mental health support and telemedicine consultations. These applications provided different services for individuals seeking non-urgent medical attention. Several government applications also emerged during this period, which prioritized health record access and administrative efficiency. The pre-pandemic era demonstrated a steady growth in mHealth, driven by private sector innovation and an increasing demand for remote healthcare. However, based on our review of these mHealth applications, they have limited functionality and significant usability challenges. The majority of users of such apps struggle to value and also adopt these tools due to poorly designed interfaces, inadequate support, and lack of features, which is highlighted by the low application ratings on the different app stores (Table 1). This period reflected a fragmented digital healthcare ecosystem, with applications operating in silos and lacking the integrated approach needed to address the diverse healthcare requirements of the population.

However, the COVID-19 pandemic marked a pivotal shift in mHealth, with the focus transitioning from individual health services to public health solutions. Government-led initiatives surged, reflecting the urgency of addressing the pandemic’s challenges. Applications for contact tracing, vaccination management, and pandemic monitoring became central to national health strategies. In addition to public health measures, telemedicine platforms experienced unprecedented demand as they provided safe and remote alternatives to in-person healthcare. This period demonstrated how digital health solutions could rapidly adapt to large-scale health crises, emphasizing the critical role of government and private sector collaboration in deploying widespread, accessible technology for health interventions. The pandemic highlighted the role of mHealth as enablers of public health, ensuring continuity of care and improving accessibility during crises. On the other hand, the mHealth landscape post-pandemic in Saudi Arabia has transitioned toward creating comprehensive digital health ecosystems and addressing long-term healthcare challenges while building on the digital foundations established during the pandemic (Figure 2). Applications that were once narrowly focused on pandemic management have evolved into multifaceted platforms, integrating a broad range of healthcare services into unified interfaces. For example, *Tawakkalna*, initially designed for contact tracing, now includes features such as medical appointment scheduling, telemedicine consultations, telepharmacy, medical record access, and administrative tools, reflecting its transformation into a *“super-app”* for healthcare and beyond. Similarly, the *Sehhaty* application has integrated functionalities from multiple government mHealth applications that were introduced during the pandemic (i.e., *Mawid*, *Seha*, and *Tetamman*), offering telemedicine consultations, electronic prescription management, sick leave request, and health tracking. This shift toward integrated platforms represents a deliberate effort to address the fragmented nature of the pre-pandemic mHealth ecosystem while building on the widespread adoption driven by the pandemic.

Despite these advancements, significant challenges remain. Issues related to usability and accessibility still exist, particularly in navigating increasingly complex user interfaces. While developers have made strides in improving user interfaces, the persistence of digital literacy gaps also limits the ability of many users to use these tools effectively. Additionally, privacy and trust concerns remain a critical issue. Addressing these concerns is essential for fostering confidence in mHealth applications, particularly among users who may be more hesitant to share sensitive information. The evolution of mHealth applications across these three phases highlight the dynamic interplay between technological innovation and the societal context in which it occurs. The pandemic acted as a catalyst, driving rapid advancements while simultaneously exposing systemic inequities that must be addressed in the post-pandemic era. The move toward integrated, user-centered platforms reflects a maturing digital healthcare ecosystem, but the ongoing challenges faced by users highlight the need for continued focus on inclusivity and accessibility. The lessons learned from this trajectory provide valuable insights for creating equitable and effective mHealth solutions that meet the needs of all users.

**Figure 3:**
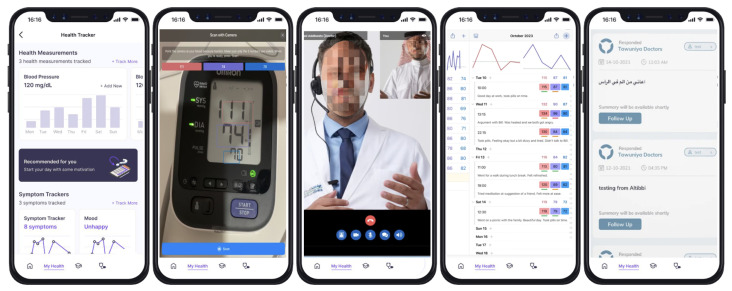
Interfaces of various mHealth applications in Saudi Arabia showcasing diverse features, including doctor selection and availability, consultation records, and virtual video consultations.

The analysis of mHealth applications in Saudi Arabia highlights the diversity and breadth of features offered by existing platforms, alongside notable gaps and opportunities for future development (Figure 3). This study identified a range of core functionalities, including doctor search, appointment booking, telemedicine, and health education, which are widely implemented across the applications. However, the depth of feature integration varies significantly among the platforms, with some applications demonstrating comprehensive offerings, such as Sehhaty, while others provide limited capabilities. The resulting matrix (Figure 2) highlights significant disparities in feature coverage, with core functionalities such as appointment booking (86%) and telemedicine (76%) being widely implemented, while features like health device integration (24%) and symptom checker (19%) remain underutilized. The percentages in the bottom row represent the proportion of features implemented by each application, ranging from 97% in Dr. Sulaiman Al Habib to 18% in Health Voluntering. This analysis provides insight into the strengths and gaps in mHealth application functionalities in Saudi Arabia. Functionalities such as health device integration, digital support groups, and family health management are notably underrepresented, with adoption percentages remaining below 50% across most applications. This indicates a potential area for growth, as these features could enhance user engagement and address the increasing demand for personalized healthcare solutions. Additionally, emerging technologies such as AI-enabled symptom checkers and gamified wellness demonstrate limited availability, suggesting an opportunity to improve preventive healthcare measures through digital platforms.

Our findings reveal that while mHealth applications in Saudi Arabia have successfully adopted essential features, there remain significant gaps in innovation and user-centric functionalities. The lack of integration for expense management, insurance claims, and online payments reflects a need for better financial management tools within these platforms. Future opportunities include the development of comprehensive mHealth ecosystems that unify core healthcare services with advanced, user-friendly features. These enhancements would not only bridge the current feature gaps but also support Saudi Arabia’s broader vision of a digitally connected healthcare landscape that prioritizes accessibility, personalization, and proactive health management. A deeper examination of the feature distribution reveals specific strengths and weaknesses within individual applications, as well as broader trends across the mHealth landscape in Saudi Arabia. For example, Dr. Sulaiman Al Habib stands out with 97% of feature coverage, reflecting its focus on providing comprehensive healthcare services. These platforms include widely used features such as doctor search, appointment booking, telemedicine, and medical record access, making them highly versatile for users seeking both basic and advanced healthcare functionalities. Similarly, applications like Sehhaty, NahdiCare Clinics, and Meena Health excel in telemedicine services, which are critical in enhancing remote access to healthcare professionals. On the other hand, platforms like Labayh emphasize health education and mental health consultations, addressing niche areas within the healthcare spectrum.

The resulting matrix highlights a clear opportunity for applications to focus on preventive care, on-demand symptom checkers, healthcare management, and financial management tools. Features like expense management, insurance claims, and split payments are absent in more than 38% of applications, indicating a noticeable gap in addressing the financial complexities of healthcare. Similarly, wellness programs and symptom checkers, which can empower users to take a proactive role in their health, remain underdeveloped across most platforms. The variations in feature integration suggest that while some applications are excelling in specific areas, such as telemedicine or health education, others lack the versatility to address a comprehensive range of user needs. Future development efforts should aim to create integrated solutions that combine essential features with advanced tools for personalized healthcare. For example, integrating emergency alerts, wearable device support, and holistic family health management would significantly enhance the user experience and meet the diverse needs of Saudi Arabia’s healthcare users. By addressing these gaps and opportunities, mHealth applications can not only improve their functionality but also align more closely with the needs of the users, improving overall healthcare accessibility for all.

The growing government-led efforts in Saudi Arabia, particularly through unified digital health platforms such as Sehhaty and Tawakkalna, which integrate essential services like telemedicine, vaccination records, and appointment booking, reflect a broader global shift toward patient-centered mHealth solutions. Similarly, countries such as the United Kingdom [38] and the United Arab Emirates [39] have developed mHealth interventions and integrated digital health ecosystems that offer comprehensive access to public health services through centralized platforms. These parallels highlight a shared commitment across nations to enhancing healthcare accessibility, coordination, and continuity through digital innovation [40].

The distribution of feature coverage mapped against the WHO digital health classification reveals an interesting pattern in the priorities and gaps of current digital health applications. Features categorized under **WHO 1.0 Persons**, which focus on empowering individuals with tools for self-care, access, and engagement, show a moderate implementation rate of 60% across the reviewed applications. Within this domain, certain features like appointment booking (86%), health education (91%), and medication management (81%) are widely adopted, suggesting that many platforms prioritize core interactions that facilitate access to services and health literacy. Similarly, medical record access and customer support are also at 86%, indicating that administrative convenience and communication are central to user experience. In contrast, features such as symptom checkers (19%) and digital support groups (10%) are severely underrepresented, highlighting a notable neglect of personalized, real-time support, and community-building elements which are critical for proactive health engagement and chronic condition management.

In contrast to other categories, the matrix appears significantly more fragmented within the **WHO 2.0 Providers** category. This category shows the lowest average implementation at just 40%, driven to its decline by the near absence of features like hospital check-ins (10%), wait time estimation (10%), and digital queues (14%). These are pivotal functionalities for streamlining patient flow and improving care efficiency, and their limited presence suggests that most platforms are not yet leveraging digital tools to optimize provider operations. While telemedicine and consultation (both 76%) reflect some investment in remote care capabilities, these findings highlight a reactive, rather than systemic, integration of features.

In contrast, the **WHO 3.0 Management** category, with an average coverage of 63%, presents a relatively balanced profile. The 100% adherence to credibility measured across all mHealth applications in this study reflects the regulatory obligations imposed by the national governance structure under which these platforms operate. All 21 applications are either government-owned or government-approved, which subjects them to strict compliance with the Saudi Personal Data Protection Law (PDPL) [41]. The PDPL mandates robust safeguards for handling personal data, including principles of transparency, accountability, user consent, and secure data processing cornerstones of digital credibility. As a result, adherence to credibility standards is not merely a design choice but a legal requirement. This regulatory alignment ensures that all included platforms follow standardized measures that foster user trust, protect sensitive health information, and maintain the integrity of digital health services. Furthermore, family health management (81%) and expense management (62%) also show solid integration, highlighting the growing emphasis on supporting everyday health activities and family well-being alongside core clinical services. However, features such as geo-based alerts (33%) remain limited, which suggests that real-time, context-aware functionalities are not yet prioritized in current mHealth solutions.

Finally, **WHO 4.0 Data**, which emphasizes interoperability, privacy, and security, has the highest average implementation at 66%, driven by high adoption rates of medical record sharing (91%), privacy measures (76%), and two-factor authentication (76%). This suggests a growing awareness of data integrity and protection as a foundational requirement in digital health. Still, the integration of health devices (24%) and biometric login (47%) lag behind, revealing an underutilization of advanced personal monitoring and seamless identity verification technologies. Together, these findings expose a digital health ecosystem, where user-centric administrative tools are relatively mature, but deeper systemic integration, provider workflow enhancement, and advanced personalization remain limited. The alignment with WHO classifications not only highlights the functional strengths and weaknesses of current mHealth applications but also serves as a framework for guiding future development toward more holistic and patient-centered digital health ecosystems.

The observed variability in feature adoption across mHealth applications in Saudi Arabia highlight the fragmented maturity of digital health integration. While a few platforms demonstrate a robust breadth of functionality, the majority reflect partial or uneven implementation. This divergence suggests that digital health development is not proceeding under a unified design paradigm but is instead shaped by decentralized operational priorities, varying funding levels, and differing assumptions about user needs. Such inconsistency presents a challenge to user continuity and expectations; for example, a patient accustomed to accessing medical records and booking appointments through one app may find critical functions missing in another. This nonuniformity in feature availability creates a disjointed user experience, which could undermine trust, limit adoption, and ultimately fragment the benefits of digital health investments.

### Design and Practical Implications

The analysis of mHealth applications in Saudi Arabia reveals critical insights into both the current design practices and the strategic directions needed for future development. While many apps demonstrate strong functionality in core areas such as appointment booking, telemedicine, and health record access, notable gaps persist in features related to user engagement, personalization, accessibility, and interoperability. These findings have important implications for developers, policymakers, and healthcare providers seeking to enhance the effectiveness, inclusivity, and sustainability of digital health tools.

***User-Centered Design:*** Prioritizing user experience and usability by adopting a user-centered design approach is essential to ensure that mHealth applications remain intuitive, accessible, and accommodating to diverse user demographics, especially individuals with varying levels of technological literacy [42,43,44]. This may include incorporating larger font sizes, voice commands, and visual cues to simplify navigation, as well as designing workflows that minimize cognitive load [45]. Prior studies have demonstrated that usability testing often reveals overlooked barriers, such as the need for simplified authentication processes or offline functionality for users in regions with unreliable internet [46,47]. Also, collaborating with public health authorities and medical experts is essential in designing mHealth applications that align with best practices and integrate seamlessly into existing public health infrastructures. Engaging healthcare professionals in the development process ensures that these applications are clinically relevant, user-friendly, and capable of addressing real-world health challenges [48]. Such partnerships facilitate the creation of standardized protocols, enhance data interoperability, and promote the adoption of mHealth solutions within healthcare systems [49,50].

***A Shift from Reactive to Proactive mHealth Strategies:*** The evolution of mHealth applications in Saudi Arabia reveals a transformative journey that is as much about technological advancement as it is about addressing socio-cultural and systemic barriers. This study sheds light on the broader implications for healthcare delivery and digital inclusivity in a rapidly digitizing society. The COVID-19 pandemic acted as a catalyst, pushing mHealth applications to the forefront of healthcare delivery [4]. Initially, these applications were reactive, addressing immediate public health needs such as contact tracing, vaccination management, and pandemic awareness through tools like Tawakkalna and Tabaoud. However, as the pandemic subsided, mHealth applications evolved into proactive, comprehensive platforms aimed at holistic healthcare management. The transition from niche, pandemic-driven functionalities to broader, integrated services exemplifies the adaptability of digital healthcare to shifting societal needs. Despite initial challenges, including usability issues and reliance on external assistance, users have increasingly recognized the potential of these tools in improving their quality of life [27]. Applications like *Sehhaty* signify a paradigm shift toward unifying services under a single, accessible interface. This progression highlights the importance of designing mHealth applications that not only address immediate crises but also sustain long-term health management goals.

***A Shift Toward Integrated Services:*** Fragmentation within the mHealth ecosystem emerged as a consistent challenge in the findings, with users often struggling to navigate multiple applications for different healthcare needs. To address this, mHealth applications must transition toward integrated service models that consolidate functionalities into unified, holistic solutions [40,51]. For instance, a single application could offer features such as appointment scheduling, telemedicine consultations, medication reminders, and integration with home health devices for health tracking. This integration reduces the need for multiple access and logins, minimizes cognitive strain, and streamlines access to healthcare services. Moreover, linking mHealth tools with government databases and existing healthcare systems ensures that users have seamless access to their medical history and can benefit from coordinated care. By adopting an integrated approach, developers can not only improve the usability of mHealth applications but also enhance their effectiveness in delivering comprehensive healthcare solutions to diverse populations.

***A Shift Toward a Unified Design Framework for Digital Health:*** Given the observed fragmentation in functionality across mHealth applications, there is a compelling need for a centralized digital health design framework that ensures baseline consistency in user experience, service availability, privacy, and accessibility. Such a framework would define a standardized set of essential features, such as appointment booking, medical record access, telemedicine, and privacy safeguards, that all mHealth applications must implement or support. While prior frameworks have primarily focused on post-development evaluation and regulatory compliance, they often fall short in providing comprehensive, design-oriented guidance tailored to digital health [32,52,53]. Proposing a unified design framework would serve as both a regulatory guide and a design blueprint, promoting interoperability, reducing redundancy, and fostering user trust across platforms. By mandating adherence to a common set of core features, such a framework would ensure that no matter which application a user uses, they receive a consistent and reliable standard of digital healthcare. Moreover, it would streamline development efforts, support integration with national health ecosystems, and accelerate the digital maturity of platforms. Such a framework represents a strategic step toward achieving equitable and unified digital health service delivery at scale. This study and the resulting feature matrix can serve as a foundational basis for informing and shaping such a unified design framework.

## 5. Limitations

While this study offers valuable insights into the landscape of mHealth applications in Saudi Arabia, it is important to acknowledge its methodological limitations. These limitations are discussed in terms of internal and external validation to clarify the boundaries of the study’s findings and their broader applicability.

**Internal Validation.** To ensure the validity and trustworthiness of our qualitative analysis, multiple strategies were employed throughout the research process. First, methodological transparency was maintained by clearly outlining the coding procedures and thematic development stages, allowing for replicability. Second, this study utilized data drawing from app store descriptions, official app documentation, and publicly available institutional information to enhance the credibility and robustness of the thematic findings. Third, an audit trail was maintained in ATLAS.ti to systematically document coding decisions, theme revisions, and data interpretation steps. Finally, the main features and deficiencies of each application were thoroughly examined by the researcher, with close attention to their usability, functionality, and alignment with digital health standards.

**External Validation.** Despite the internal rigor, the generalizability of the findings may be limited. The analysis focused exclusively on mHealth applications available and accessible within the Saudi Arabian context, which may reflect region-specific regulatory and healthcare infrastructure considerations. As such, the presence or absence of certain features may not translate directly to other countries or health systems. Additionally, because this study was based on publicly observable app functionalities rather than direct developer input or user data, some advanced or backend features may have been underrepresented. Future research incorporating user feedback, clinical validation metrics, and longitudinal app performance data would enhance the external validity and applicability of these findings across diverse contexts.

## 6. Conclusions and Future Work

This study provides an overview of the current landscape of mHealth applications in Saudi Arabia, identifying their core features, gaps, and opportunities for improvement. The findings highlight that while mHealth applications in the region have successfully implemented essential functionalities such as doctor search, appointment booking, and telemedicine, advanced features like health device integration, health management, and symptom checkers remain underutilized. These gaps present significant opportunities for developers to enhance the scope and usability of these platforms, thereby improving access to personalized and holistic healthcare solutions. Moreover, this study highlights the potential of mHealth applications in contributing to improve healthcare accessibility. We aim to expand on these findings by conducting user studies to explore the current perceptions, challenges, and satisfaction levels of mHealth application users in Saudi Arabia. Key areas of focus will include ease of use, reliability of features, accessibility for diverse demographics, and privacy concerns. Understanding these aspects will provide a more user-centric perspective on the current state of mHealth applications.

Another avenue for future work is to investigate the challenges users face when utilizing these platforms, such as technical barriers, lack of integration with existing healthcare systems, and limited support for chronic disease management. Addressing these challenges could inform the development of more intuitive and inclusive mHealth solutions. Also, there is a need to explore not just which features are present, but how they are being used, by whom, and with what outcomes. For instance, does the presence of multilingual support meaningfully enhance engagement among non-native speakers? Do platforms that include gamification or digital support communities show better adherence among patients with chronic conditions? Ultimately, these insights call for a new generation of user-centered, evidence-based design studies that move beyond checklist compliance and toward understanding the lived experiences and health outcomes that these digital tools are meant to support. Additionally, future research could explore the role of emerging technologies, such as generative artificial intelligence and machine learning techniques, in enhancing mHealth applications. These technologies have the potential to improve personalization, predictive healthcare, and proactive health management. By incorporating such technologies, developers can create mHealth applications that are not only functionally robust but also aligned with the evolving needs of users and the healthcare ecosystem.

## Figures and Tables

**Table 1:** An overview of the mHealth applications available in Saudi Arabia that was reviewed in this study. The list shows the app name, release date, main service provided, type of app, functional role classification, and app store ratings.

Application Name	Date	Main Service	Type	Functional Role	Rating
Cura	2016	Health Services	Private App	Care Delivery	1.5
Altibbi	2018	Telemedicine	Private App	Care Delivery	4.4
Labayh	2018	Mental Health	Private App	Support/Wellness	4.6
Fakeeh Care	2019	Health Services	Private Hospital	Care Delivery	4.6
Dr. Sulaiman Al Habib	2019	Health Services	Private Hospital	Care Delivery	4.1
MNGHA Patient Care	2019	Health Services	Gov. Hospital	Care Delivery	2.8
iKFMC	2019	Health Services	Gov. Hospital	Care Delivery	3.7
Bupa Arabia	2019	Insurance	Insurance App	Administrative	4.4
My Clinic KSA	2020	Health Services	Private Clinic	Care Delivery	3.9
NahdiCare Clinics	2020	Health Services	Private Clinic	Care Delivery	4.5
Sanar	2020	Telemedicine	Private App	Care Delivery	4.7
Tawakkalna	2020	Health Tracking	Gov. App	Management	4.2
Wateen	2020	Blood Donation	Gov. App	Support/Wellness	4.0
Altakhassusi	2020	Health Services	Gov. Hospital	Care Delivery	4.8
Sehhaty	2020	Health Services	Gov. App	Management	4.6
Qariboon	2021	Community	Gov. App	Support/Wellness	3.0
Health Volunteering	2021	Volunteering	Gov. App	Support/Wellness	1.9
SFHP Riyadh	2021	Health Services	Gov. Hospital	Care Delivery	1.7
Tawuniya	2021	Insurance	Insurance App	Administrative	4.4
Al Mostajeeb	2022	Emergency	Gov. App	Management	2.8
Meena Health	2022	Health Services	Private Clinic	Care Delivery	3.8

## Data Availability

The original contributions presented in this study are included in the article. Further inquiries can be directed to the corresponding author.
